# Study on two nematode species suggests climate change will inflict greater crop damage

**DOI:** 10.1038/s41598-023-41466-x

**Published:** 2023-08-30

**Authors:** Churamani Khanal, Julian Land

**Affiliations:** 1 Department of Plant and Environmental Sciences, Clemson University, Clemson, SC 29634 USA; 2 Rheinland-Pfälzische Technische Universität, Campus Landau, Wolfsmilchweg 7, 55262 Ingelheim, Germany

**Keywords:** Microbiology, Ecology

## Abstract

Food security has become one of the greatest challenges of the millennium and it is predicted to be exacerbated by climate change due to the adverse effects of soil temperature on crop productivity. Although plant-parasitic nematodes are one of the most important limiting factors of agricultural production, the fate of soil temperature in their biology is not fully understood. Here we present the effects of soil temperature on survival, reproduction, virulence, and disease severity from the perspective of two nematode species *Rotylenchulus reniformis* and *Meloidogyne floridensis*. The two nematode species were purposefully selected to represent a significant threat to annual and perennial crops. We employed novel approaches of direct as well as indirect heat exposure to evaluate nematode biology. The direct heat exposure assay involved the exposure of nematodes to hot water in a heating block at 32, 33, and 34 °C for 7 h, and subsequent evaluation of their survival after 18 h. The indirect exposure assay employed a commercial heat mat to raise soil temperatures to 32, 33, and 34 °C for 7 h during the daytime, and subsequent evaluation of nematode reproduction, virulence, and/or disease severity over the period of 6 weeks after inoculation. When directly exposed to hot water at 34 °C, the survival of *R. reniformis* increased by 10% while the survival of *M. floridensis* decreased by 12% relative to that at 32 °C. Upon increasing soil temperatures from 32 to 34 °C, the reproduction of *R. reniformis* and *M. floridensis* decreased by 49% and 53%, respectively. A significant reduction in the reproduction of *M. floridensis* occurred when soil temperature was increased from 33 to 34 °C, however, the same condition did not significantly affect *R. reniformis* reproduction suggesting the latter species has a greater ability to adapt to increasing soil temperature. Additionally, the virulence of *R. reniformis* was greater at 33 and 34 °C relative to that at 30 °C indicating increased aggressiveness of the nematode at higher soil temperatures. The virulence of *M. floridensis* appeared to be decreased as evident from increased root biomass when soil temperature was increased from 32 to 34 °C, however, the greater root biomass may have resulted from increased root galling at the higher temperatures. Results of the current study suggest that while higher soil temperatures due to climate change may lead to reduced nematode reproduction, crop losses will likely increase due to increased nematode virulence. Through the current study, we report practical evidence of the quantitative impact of climate change on the biology of plant-parasitic nematodes. Further studies involving a wider range of temperature and exposure time are needed to better understand nematode biology under climate change.

## Introduction

Plant-parasitic nematodes (PPN) are microscopic non-segmented pseudocoelomic worms that are cosmopolitan in distribution and feed on plants as obligatory pathogens. More than 4000 species of PPN have been described, and they are responsible for approximately $358 billion in crop losses worldwide every year^1^. Among several species of PPN, the reniform nematode (*Rotylenchulus reniformis*) is one of the most economically important nematode species specifically in the US where approximately 168 thousand bales of cotton and 2 million bushels of soybean are lost to this nematode annually^2,3^. The presence of as few as two *R. reniformis* in a cubic centimeter of soil early in the crop growing season is enough to elicit economic crop loss by the end of the crop growing season^4^. Similarly, a new and emerging peach root-knot nematode (*Meloidogyne floridensis*) is becoming an increasing threat to tree crop industries in the US as management options available for other species of *Meloidogyne* are not effective against this nematode^5^. Management of these nematodes will continue to be a challenge because of (i) the lack of host-plant resistance or preference of growers towards high-yielding cultivars with little to no regard to resistance status, (ii) existence of significant variability in reproduction and virulence among geographic isolates of nematodes leading to lower performance of resistant lines^6–9^, (iii) inability of nematicides to provide season-long protection against nematodes^10^, (iv) adverse effects of fumigants on the environment and human health^7^, and (v) lack of consorted efforts among researchers and policy makers to combat crop losses to nematodes in the future with warmer soils.

The average global temperature is rising at a pace the planet has not experienced in millennia, with the last 8 years being the warmest^11^. A recent study that analyzed a 1170-year-long tree-ring anatomy suggested the current climate is substantially warmer than that of the medieval period^12^. Additionally, the Intergovernmental Panel on Climate Change’s Sixth Assessment Report from 2023 highlighted that the global temperature has increased by 1.09 °C since the late nineteenth century and is predicted to rise 1.5 °C by 2040 and 4.4 °C by 2100 (https://report.ipcc.ch/ar6syr/pdf/IPCC_AR6_SYR_SPM.pdf). The most significant impact of climate change will be on agriculture as warmer environments will pose physiological stress to plants and will lead to increased insect and disease pressure^13^. In addition to the gradual rise in soil temperature, climate change induced heat waves that are occurring more frequently and for prolonged time lead to greater crop losses suggesting assessment of impact of climate change requires more careful attention to prolonged exposure of plants to heat^14^. Furthermore, nematode attack to heat stressed plants may greatly alter the crop physiology leading to even greater crop losses. The world needs to be better prepared to mitigate crop losses due to climate change to sustainably feed the growing population which, according to United Nations, is expected to reach 9.7 billion by 2050.

From an evolutionary perspective, nematodes are one of the most adaptive organisms on earth as they evolved from free-living marine species to sedentary endoparasites^15,16^. Having their ability to co-evolve with the environment, nematodes will likely continue to adapt themselves to the changing environment so they can continue to colonize the plants. Provided nematodes can colonize plants in the same or a better way in warmer soils, the crop lost to the nematodes in the future would likely be much higher than the currently estimated amount.

The temperature has a direct effect on nematode reproduction and other biological processes. Although optimal temperature requirement for the development can greatly vary among nematode genera and species, thermal time relationship studies in the past suggested the thermal optima to be in a range of 24–37 °C^17–19^. Additionally, nematode life cycle is shortened as the temperature is increased until it reaches the maximum level^20–24^. A shorter nematode life cycle allows nematodes to reproduce faster (increased rate of reproduction) and causes more significant damage to crops (increased virulence and disease severity). While the studies on impacts of soil temperature on nematode biology were initiated in early 1930s and were subsequently led by researchers primarily in Europe and UK, these studies were either conducted in growth chambers with constant ambient temperatures or measured soil temperatures in relation to the ambient temperature^19,25–28^. In order to develop effective nematode management programs under higher soil temperatures brought by climate change, understanding the changes in nematode biology and its consequence in plants under soil environments very similar to the natural environment is critical. Therefore, the main objective of this study was to understand the impacts of warmer environments on nematode biology regarding nematode survival, reproduction, virulence as well as disease severity from the perspective of two nematode species, *R. reniformis* and *M. floridensis*. The two nematode species were purposefully selected in this study to represent a significant threat to annual and perennial crops.

## Results

### Effects of temperature on nematode survival

**Figure 1:**
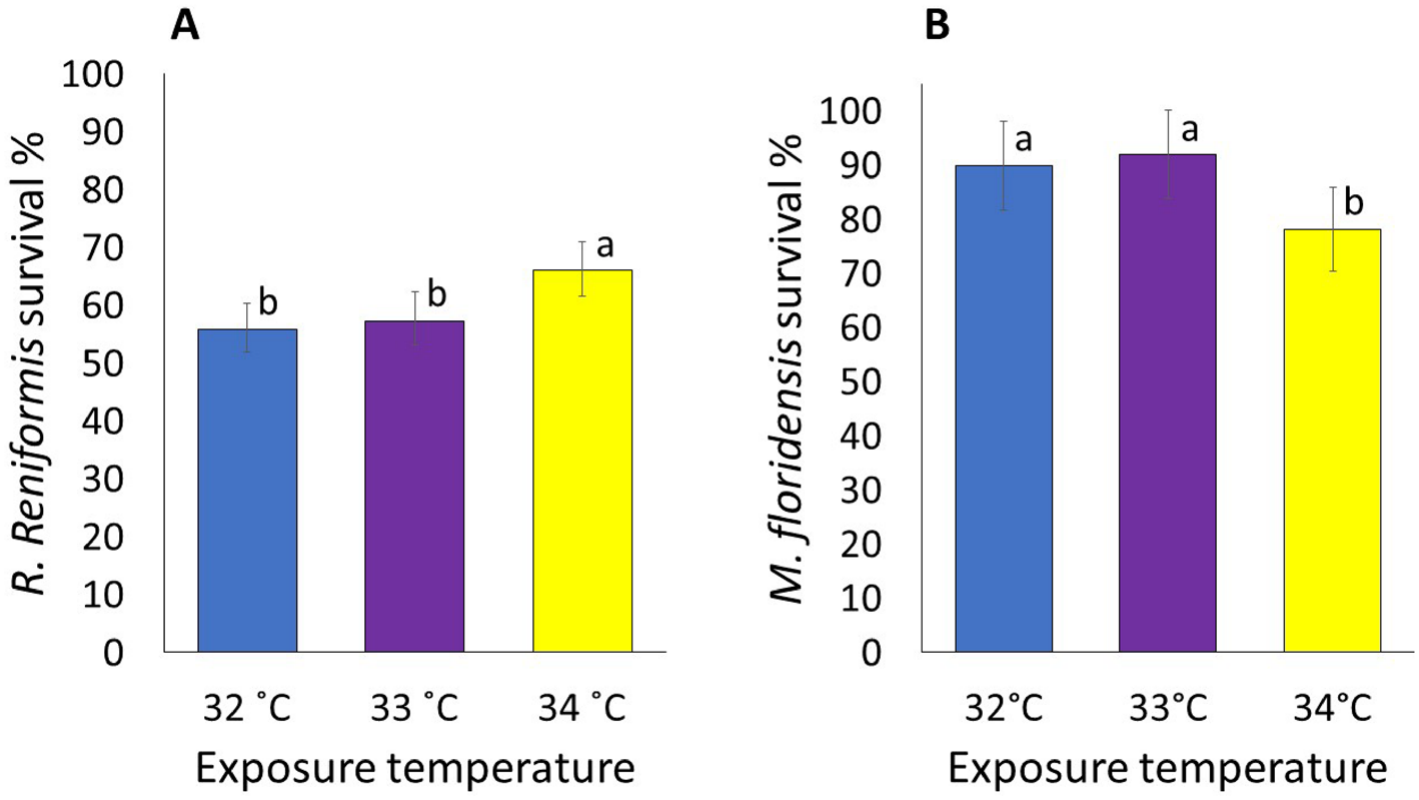
The survival of *Rotylenchulus reniformis* (**A**) and *Meloidogyne floridensis* (**B**) when directly exposed to various water temperatures. Data for *R. reniformis* were combined over four experiments as means of 20 replications. Data for *M. floridensis* were combined over two experiments as means of 10 replications. Treatment means followed by a common letter are not significantly different according to Tukey’s HSD test (*P* ≤ 0.05).

There was a significant impact of direct exposure of heat on the survival of *R. reniformis* (*P* < 0.01, Fig. 1A). The nematode survival ranged from 56 to 66%. While the least proportion of *R. reniformis* survived at 32 °C (control), the survival increased as the exposure temperature increased. At 33 °C, the survival of *R. reniformis* increased by 1%, although the increment was not significantly greater than that of the control. The survival of *R. reniformis* significantly increased at 34 °C relative to that of the control, with the increment being 10%.

The survival of *M. floridensis* was significantly impacted by the temperature (*P* < 0.01, Fig. 1B). The nematode survival was 90% at 32 °C, which increased by 2% as the temperature was increased to 33 °C, although the increment was not significant. At 34 °C, the nematode survival was down to 78% representing a significant 12% reduction relative to the control.

### Effects of temperature on reproduction of nematodes

**Figure 2:**
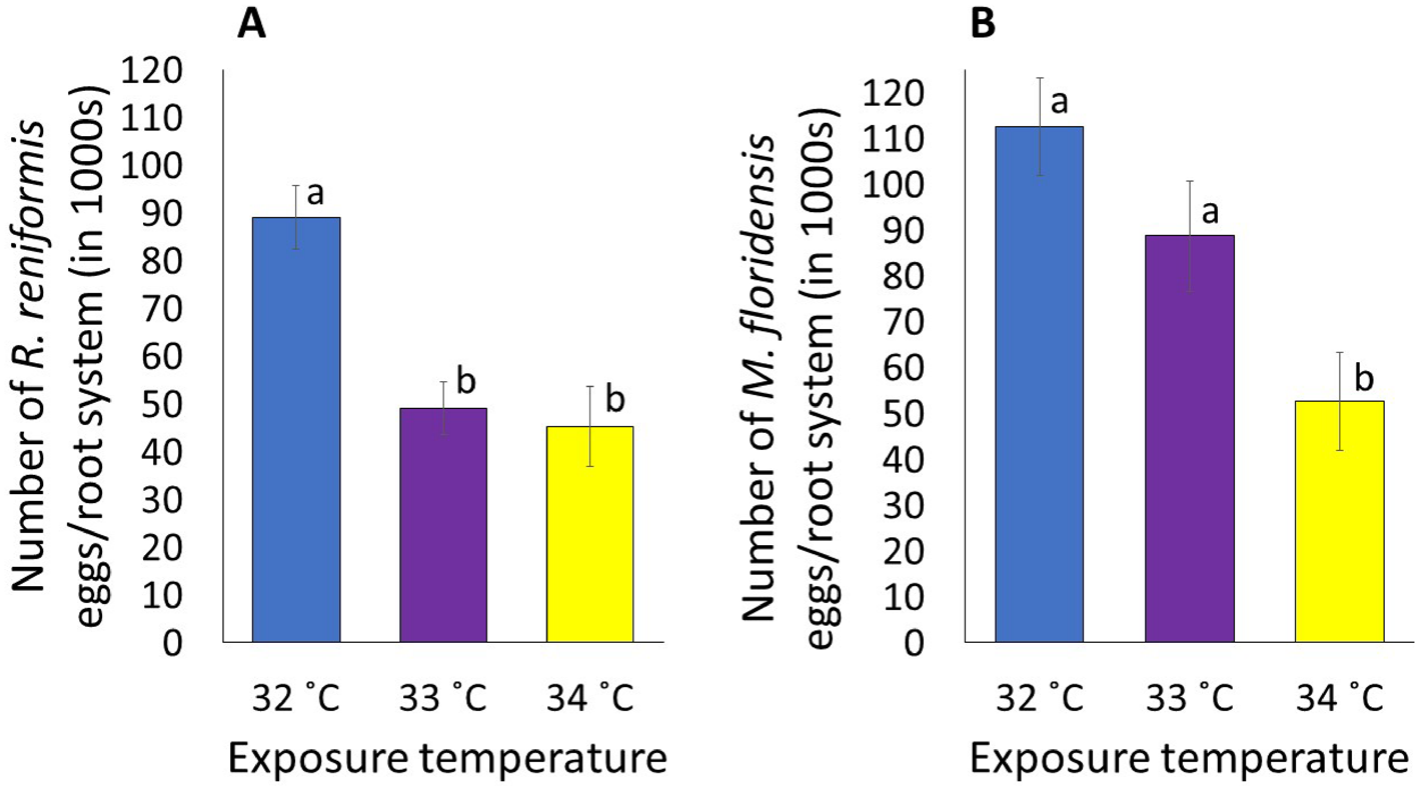
The reproduction of *Rotylenchulus reniformis* (**A**) and *Meloidogyne floridensis* (**B**) on tomato expressed as number of eggs/root system as influenced by various soil temperatures. Data were combined over two experiments and are means of 10 replications. Treatment means followed by a common letter are not significantly different according to Tukey’s HSD test (*P* ≤ 0.05).

Soil temperature significantly impacted the reproduction of *R. reniformis* (*P* = 0.01, Fig. 2A). The reproduction of *R. reniformis* across all temperatures ranged from 45,416 to 89,080 eggs/root system with the greatest reproduction occurring at 32 °C and the least reproduction occurring at 34 °C. The reproductions at 33 °C and 34 °C were significantly lower than the control, the reductions being 45% and 49%, respectively. The reproduction of *R. reniformis* at 34 °C was lower than at 33 °C, although at the same level of statistical significance.

There was a significant impact of soil temperature on the reproduction of *M. floridensis* (*P* = 0.01, Fig. 2B). The reproduction across all temperatures ranged from 52,616 to 112,568 eggs/root system with the greatest reproduction occurring at 32 °C and the least reproduction occurring at 34 °C. Although not statistically significant, a 21% reduction in reproduction occurred at 33 °C compared to the control. The reproduction at 34 °C was significantly reduced, which was 41% lower than that at 34 °C and 53% lower than the control.

### Effects of temperature on virulence of nematodes

The root and shoot biomass of tomatoes infected with either nematode species did not appear to be significantly impacted by increasing soil temperature (Table 1). However, there were numerical differences in root and shot biomass at various temperatures indicating the impact of soil temperature on virulence of nematodes. The root and shoot biomass of the plants infected with *R. reniformis* at 34 °C was reduced by 23% and 8%, respectively, relative to that at the control temperature. Compared to the control, the root biomass of tomato infected with *M. floridenis* at 34 °C was increased by 6% while the shoot biomass was decreased by 9%.

**Table 1:** The virulence of *Rotylenchulus reniformis* and *Meloidogyne floridensis* on tomato at various soil temperatures expressed as total root and shoot biomass upon nematode infection.

Exposure temperature	*R. reniformis*		*M. floridensis*	
	Dry root wt. (g)	Dry shoot wt. (g)	Dry root wt. (g)	Dry shoot wt. (g)
32 °C	1.20 a	6.16 a	1.02 a	4.87 a
33 °C	0.88 a	5.87 a	0.93 a	5.38 a
34 °C	0.92 a	5.70 a	1.08 a	4.44 a
*P*-value	0.3136	0.9680	0.9231	0.7315

Data for each nematode were combined over two experiments and are means of 10 replications. Treatment means followed by a common letter within a column are not significantly different according to Tukey’s HSD test (*P* ≤ 0.05).

### Effects of temperature on disease severity

**Figure 3:**
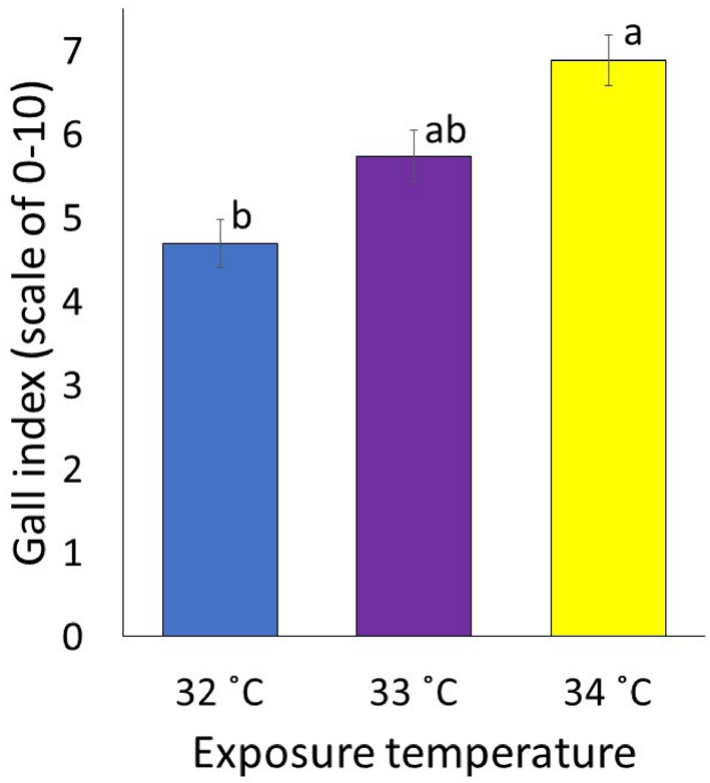
The disease severity of *Meloidogyne floridensis* on tomato expressed as root gall index (scale of 0 to 10, where 0 = no galls and 10 = completely galled roots) as influenced by various soil temperatures. Data were combined over two experiments and are means of 10 replications. Treatment means followed by a common letter are not significantly different according to Tukey’s HSD test (*P* ≤ 0.05).

Disease severity by *M. floridensis* on tomato was significantly impacted by the soil temperature (*P* = 0.01, Fig. 3). The root gall index continued to increase as the soil temperature increased. A 22% increase in gall index was observed at 33 °C, although not significantly higher than the control. The root gall index was significantly increased at 34 °C relative to the control, with the increment being 46%.

## Discussion

As the world soil temperature continues to increase due to climate change, it will likely alter the biology of PPN. While PPN belong to the oldest living organisms on earth^29,30^ and they have been successful in co-evolving with the changing environment over the past millions of years^31^, it is not clear whether their ability to damage crops will remain the same or increase in the currently changing environment. Additionally, as the effect of climate change is not uniform across the globe, some regions may experience extreme weather such as heat waves which can have further consequences in nematode biology. The current study was employed to understand the impact of direct exposure of nematodes to hot water as a simulation of heat waves, as well as the impact of indirect heat exposure as a simulation of rising soil temperatures over the next several decades. Results from the direct heat exposure assays suggest that the survival of *R. reniformis* will significantly increase in the event of heat waves while the survival of *M. floridensis* will significantly decrease. While the current study is the first of its kind to evaluate the effect of direct heat exposure on *R. reniformis* and *M. floridensis*, a handful of studies have been conducted in the past to assess the effect of hot water on other nematode species. A study evaluated the effect of hot water at various temperatures (40–52 °C) for various exposure times (1 min to 4 h) and found that mortality of *M. hapla*, *Pratylenchus penetrans,* and *Aphelenchoides besseyi* was positively correlated with water bath temperature and exposure times^32^. Another study also reported an increase in mortality of *M. enterolobii* as the water temperature and exposure times were increased^33^. A review of the literature suggests that optimal temperatures for the development and reproduction of *R. reniform* range from 30 to 37.5 °C^34–36^. Additionally, *R. reniformis* can enter into the state of anhydrobiosis^37^, an indication that this nematode has an inherent ability to withstand adverse environmental conditions such as drought and heat. Based on the findings of previous studies as well as the current study, it is imperative that climate change induced heat waves are likely to be detrimental to the survival of *Meloidogyne* and many other nematode species while it will likely benefit *R. reniformis* leading to their increased survival.

The nematode reproduction was decreased as the soil temperature was increased, however, contrasting differences were observed between the two nematode species in terms of the extent and rate of impact. When the soil temperature increased from 32 to 33 °C, the reproduction of *R. reniformis* sharply declined at a significant level while only a numerical decrease in the reproduction of *M. floridensis* was observed. The reproduction of *R. reniformis* slightly decreased as the soil temperature was increased to 34 °C, which was statistically similar to that at 33 °C. The reproduction of *M. floridensis*, however, sharply decreased at a significant level when the temperature was increased to 34 °C. The results suggest that *M. floridensis* is likely to greatly reduce its reproduction ability when soil temperatures go beyond 34 °C while the *R. reniformis* is likely to maintain its reproduction at a steady rate; however, further studies are needed to validate this assumption. The assumption of the ability of *R. reniformis* to thrive well in higher soil temperatures is supported by the evidence from previous studies that mention their optimal reproduction at as high as 37.5 °C^34–36^. Furthermore, up-regulation of stress-induced genes upon mild repeated heat treatments early in life has been found to enhance stress response and increased the lifespan of nematodes, flies, and human fibroblasts^37–39^. Studies on *C. elegans* suggest the increase in lifespan is associated with elevated expression of heat shock proteins and other stress response genes^38,40–43^. Studies are needed to determine if *R. reniformis* upregulates the stress-induced genes upon heat treatments leading to their increased survival, reproductive ability, and subsequent virulence. Furthermore, studies are needed to understand whether the upregulation of heat-induced genes is transient or it lasts throughout the life of the nematode for a larger range of temperatures.

Understanding the virulence of nematodes is extremely important to predict the extent of damage they can cause to a plant. The current study shows the virulence of the two nematode species was differentially impacted by soil temperatures. For the plants infected with *R. reniformis*, the overall root and shoot biomass decreased when soil temperature increased from 32 to 34 °C suggesting a greater virulence of *R. reniformis* at higher soil temperatures. Upon comparing the biomass of plants infected with *M. floridensis* between 32 and 34 °C, the shoot biomass was found to decrease while the root biomass was increased at the higher temperature suggesting virulence can vary among nematode species at various soil temperatures. While, to the best of our knowledge, the current study is the first to show the differential impact of soil temperatures on the virulence of *R. reniformis* and *M. floridensis*, previous studies have reported the existence of virulence phenotypes in *R. reniformis* meaning a substantial variability in reproduction and virulence is present in geographic isolates^8,44^. Although any of the previous studies did not mention possible reasons behind the existence of virulence phenotypes, the difference in soil temperature may have influenced the biology of *R. reniformis*. It seems reasonable to predict increased crop losses at higher soil temperatures due to increased nematode virulence; however, further studies are needed to determine whether the soil temperatures beyond the limit of the current study will lead to a further increase in virulence of the nematodes. Furthermore, as higher temperatures adversely affect plant growth and development^45–47^, altered plant physiology may have contributed to increased nematode virulence and disease severity at higher temperatures.

The current study found the root gall index, a measure of disease severity of root-knot nematodes, is positively correlated with soil temperature. While the reproduction of *M. floridensis* was significantly decreased as the soil temperature increased from 32 to 34 °C, the disease severity was significantly increased. This suggests that *M. floridensis* becomes more aggressive at higher temperatures leading to increased crop losses. It now makes sense that the greater root biomass of plants infected with *M. floridensis* as mentioned in the previous paragraph was the result of greater root galling at the higher temperature. Because root galls are the result of hypertrophy of the cells infected with root-knot nematodes, increased galling at the higher temperature resulted in increased root biomass. Further studies are needed to understand the relationship between nematode reproduction, virulence, and disease severity involving a greater range of soil temperatures for a longer duration.

Effective management of PPN is a challenge. Because chemical nematicides have been the most widely employed but the least preferred tools of nematode management, there is a need to develop safe yet highly effective chemical alternatives^7,33^. Efforts have been made to employ heat treatment in the form of hot water, heated steam, and anaerobic soil disinfestation as alternatives to chemical nematicides^48–50^. As evident from the current study, researchers should be aware of changes in nematode biology upon their exposure to higher temperatures while developing better nematode management programs. Because climate change further impacts the fate of any nematode management programs, more studies are warranted to understand the biology of various nematode species at a larger range of soil temperatures and greater exposure times.

## Methods

### Preparation of nematode inoculum

Cultures of *R. reniformis* and *M. floridensis* were maintained in a growth room environment on soybean (*Glycine max* L*.*, cv. Braxton, Dr. Mueller, Clemson University) and tomato (*Solanum lycopersicum* L. cv. Rutgers, Seedway, NY) plants, respectively at 27 ± 5 °C with a daylight of 14 h. Nematode eggs were extracted by agitating the plant roots in 10% commercial bleach for 4 min^51^. For assays involving nematode juveniles, the aqueous suspension containing eggs was placed in a modified Baermann pan at 28 °C in an incubator (VWR, Cornelius, OR), and freshly hatched juveniles were collected 24 h following incubation.

### Direct heat exposure assay

**Figure 4:**
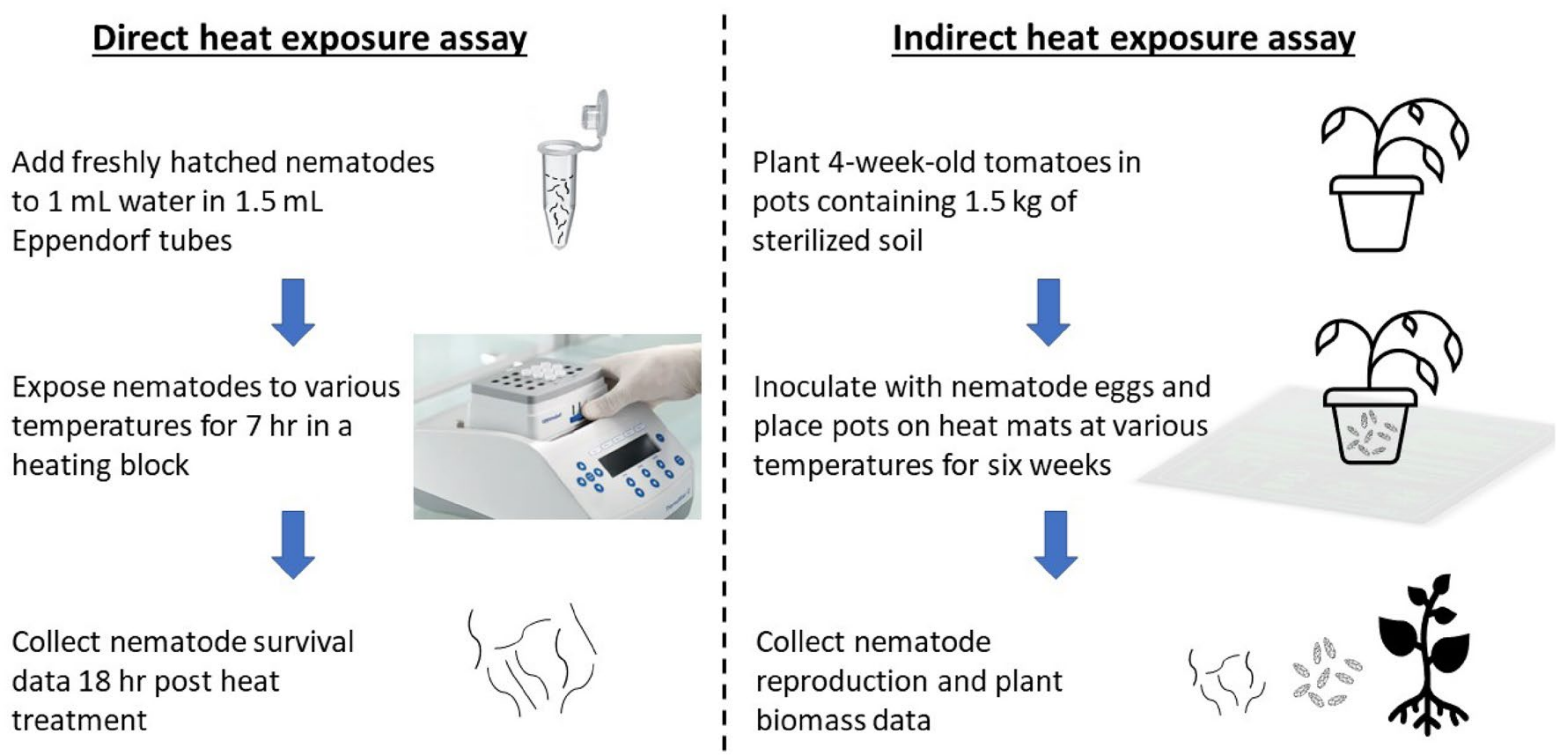
Schematic representation of direct and indirect heat exposure assays employed in the current study.

This assay was conducted to assess the impact on nematode survival upon direct exposure to heat as a close simulation of heat waves. Freshly hatched *R. reniformis* and *M. floridensis* juveniles were separately exposed to hot water at various temperatures for 7 h (Fig. 4). The water temperatures of 32°, 33°, and 34 °C, each replicated five times, served as treatment of the experiment. The lowest temperature of 32 °C was selected as a control to resemble the highest recorded soil temperature in South Carolina over the last 5 years (South Carolina State Climatology Office, 2021). The exposure duration of 7 h was selected to mimic the natural soil environment (9 am to 4 pm). Approximately 100 nematodes were placed in 1 ml of water in each 1.5 ml Eppendorf tube and the tubes were subsequently placed in Eppendorf ThermoMixer^®^ C. Nematode survival was evaluated using a compound microscope 18 h after the end of the heat treatment by adding 100 µl of 1N NaOH solution. Nematodes that moved within the period of examination (approx. 2 min) were considered alive while immobile ones with straight body shapes were considered dead. The experiment was repeated three times for *R. reniformis* and once for *M. floridensis*.

### Indirect heat exposure assay

This assay was conducted to assess the effects of various soil temperatures on the reproduction and virulence of *R. reniformis* and *M. floridensis*. The indirect assay employed commercial heat mats (iPower, Model No. GLHTMTCTRLV2HTMTSX2) that are typically used by commercial nurseries to raise seedbed temperature during the winter (Fig. 4). Aqueous suspensions containing freshly extracted 10,000 eggs were inoculated to 4-week-old tomato seedlings (cv. Rutgers, Seedway, NY) grown in 6-inch plastic pots into four 0.5 cm diameter × 5 cm deep depressions in a quadrangular pattern around the stem. Each pot received 1.5 kg of sandy loam soil steam sterilized for four cycles of 45 min at 123 °C prior to planting tomato seedling. Five pots were placed on each of three 1.2 m long and 0.5 m wide heat mats in a greenhouse during the study period. The temperature of each mat was optimized so the soils in the pot reach the temperature of 32, 33, or 34 °C. Each temperature represented the treatment of the experiment with 32 °C serving as the control. The experiment was established as randomized complete block design with five replications of each treatment. The heat mats were turned on at 9 am and turned off at 4 pm daily to mimic near-field soil conditions. The temperatures were monitored three times every day at 10 am, 12 am, and 2 pm using soil thermometers. Heat mat settings were adjusted when necessary and temperature data were collected at 3 pm every day. The experiment was established in a randomized complete block design. The temperature of the greenhouse during the study period was 27 ± 5 °C with a daylight of 14 h. Standard watering, fertilization, and insect management practices were conducted. The experiment was terminated 6 weeks after inoculation. The root gall index, as a measure of disease severity, was recorded for plants infected with *M. floridensis* using a scale of 0–10 (0 = no galls, 10 = completely galled)^52^. Nematode eggs were extracted by agitating the root systems in 10% commercial bleach for 4 min^51^. Eggs were enumerated at 40× magnification using a compound microscope (Martin Microscope Company, Easley, SC). Plant roots after egg extraction as well as the shoots were dried at 55 °C for 2 weeks. The entire experiment for each nematode species was repeated once.

### Data analysis

Data were subject to one-way analysis of variance (ANOVA) using the Generalized Linear Mixed Model in JMP PRO 16.2 (SAS Institute, Cary, NC). Residual analysis was performed to remove outliers if any were present. Goodness-of-Fit test was conducted using Shapiro–Wilk and Anderson–Darling test to determine if the data were normally distributed. Any non-normal data according to Shapiro–Wilk and Anderson–Darling test were fit to Poisson distribution analysis and transformed with *log* link to fulfill the assumption of ANOVA. Nematode survival, reproduction, plant biomass, and disease severity data were used as fixed effects while replication and experiment were used as random effects. Log transformed data were subsequently back transformed using *exp* function to obtain individual treatment means. Tukey’s HSD test (*P* ≤ 0.05) was used as post-hoc mean comparisons.

All methods were performed in accordance with the relevant guidelines and regulations.

## Data Availability

The datasets used and/or analysed during the current study will be available from the corresponding author on reasonable request.
